# Controllable electrical and physical breakdown of poly-crystalline silicon nanowires by thermally assisted electromigration

**DOI:** 10.1038/srep19314

**Published:** 2016-01-19

**Authors:** Jun-Young Park, Dong-Il Moon, Myeong-Lok Seol, Chang-Hoon Jeon, Gwang-Jae Jeon, Jin-Woo Han, Choong-Ki Kim, Sang-Jae Park, Hee Chul Lee, Yang-Kyu Choi

**Affiliations:** 1 School of Electrical Engineering, Korea Advanced Institute of Science and Technology (KAIST), Daejeon 34141, Republic of Korea; 2 Semiconductor R&D Center, Samsung Electronics, San #16 Banwol-Dong, Hwasung-City, Gyeonggi-Do 445-701, Republic of Korea; 3 Center for Nanotechnology, NASA Ames Research Center, Moffett Field, CA 94035, USA

## Abstract

The importance of poly-crystalline silicon (poly-Si) in semiconductor manufacturing is rapidly increasing due to its highly controllable conductivity and excellent, uniform deposition quality. With the continuing miniaturization of electronic components, low dimensional structures such as 1-dimensional nanowires (NWs) have attracted a great deal of attention. But such components have a much higher current density than 2- or 3- dimensional films, and high current can degrade device lifetime and lead to breakdown problems. Here, we report on the electrical and thermal characteristics of poly-Si NWs, which can also be used to control electrical and physical breakdown under high current density. This work reports a controllable catastrophic change of poly-Si NWs by thermally-assisted electromigration and underlying mechanisms. It also reports the direct and real time observation of these catastrophic changes of poly-Si nanowires for the first time, using scanning electron microscopy.

Poly-crystalline silicon (poly-Si) is widely used as a metallic or semiconducting thin film because it exhibits excellent and controllable conductivity, and can be conformally deposited with good uniformity at low cost. With these advantages, poly-Si film has been used extensively in various applications including flat panel displays, field effect transistors (FETs), solar cells, memory, integrated circuits, and microelectromechanical systems (MEMS)^1^,^2^,^3^,^4^,^5^,^6^,^7^. With recent trends in miniaturization, 2-dimensional poly-Si film and 3-dimensional (3-D) bulk poly-Si have recently been replaced by 1-dimensional (1-D) poly-Si nanowires (NWs). Poly-Si NW applications, such as 3-D stacked flash memory for ultra-high memory density, sensitive biosensors to detect bio-molecules, and revolutionary FET concepts, have drawn considerable attention. The range of nanowire applications is expected to widen further in the near future^8^,^9^,^10^,^11^,^12^,^13^,^14^.

However, as the cross-sectional area of poly-Si materials is scaled down, its current density is significantly increased, i.e., the area of electrical conduction is reduced. This high current density can cause unexpected problems caused by heat generation, which is known as Joule heating, including material decomposition, self-heating issues, and electrical and physical breakdown^15^,^16^,^17^,^18^,^19^,^20^,^21^,^22^. In contrast to the well-established body of research that exists for large-scale poly-Si thin films, the level of present research for poly-Si nanowires is still currently modest^23^,^24^,^25^.

In this work, we deeply investigated the electrical and thermal breakdown of poly-Si nanowires to analyze the catastrophic changes that occur under high current density. We report for the first time direct and real-time observations of these catastrophic and morphic changes in poly-Si nanowires, which were obtained using scanning electron microscopy (SEM). The underlying mechanisms governing the aforementioned phenomena were also verified by electrical measurements using a parameter analyzer, numerical simulations, and element analysis with energy dispersive x-ray spectroscopy (EDS).

## Results

### Structure of the poly-Si nanowire

**Figure 1:**
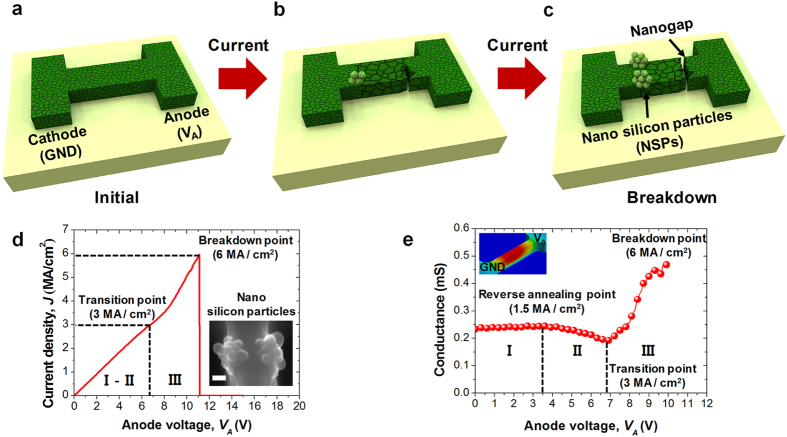
(**a**) A schematic of a poly-Si nanowire without current flowing. (**b**) A schematic of the poly-Si nanowire with high current density (5 MA/cm^2^). Grain size grows in the middle region of the poly-Si nanowire. A protruded NSPs and dented nanogap start to form near the interface where the grain size abruptly changes. (**c**) A schematic of the poly-Si nanowire when current density is further increased to 6 MA/cm^2^. The NSPs are projected and the nanogap is deepened as nano-sized grains are increased. Then physical and electrical breakdown of the poly-Si nanowire is triggered. (**d**) Measured data of applied voltage versus conduction current. After the transition point (3 MA/cm^2^), current flow is increased more rapidly than before. Inset image shows the NSPs after breakdown. (**e**) Abnormal conductance change according to the applied voltage. Inset shows simulated heat distribution profile in the nanowire, which is correlated with the gradient of the grain size change. Scale bar is 40 nm.

Poly-Si nanowires were fabricated using a top-down method (see Methods). A schematic of the test device used in this study, comprised of two probe pads located at the ends of the poly-Si nanowire, is shown in Fig. 1-a^12^,^26^. In-situ phosphorus doped poly-Si, 300 nm thick, was deposited using low pressure chemical vapor deposition (LPCVD). The doping concentration of the poly-Si was approximately 4 × 10^20 ^cm^–3^ and the average size of a grain in the fabricated nanowire was approximately 100 nm. One pad was assigned to be the anode and the other pad was assigned as a cathode, which was the ground (GND).

### Electrical and thermal characteristics of the poly-Si nanowire

Catastrophic and morphic changes, i.e., nano silicon particles (NSPs) and nanogap features, began forming in the poly-Si when the current density reached 5 MA/cm^2^, as a result of thermally assisted electromigration. A schematic of these changes is shown in Fig. 1-b. The diameter of the protruded single NSP is less than 40 nm, and it has a spherical shape (Figure S1). It should be noted that the NSPs were created at a position close to the cathode, while the nanogaps were formed near the anode. These effects occur as Si atoms that are situated near the anode drift away towards the cathode, carried by the electron wind. As a result, nanogaps and NSPs are created near the anode and cathode, respectively. If more current flows through the nanowire, the diameter of the NSP rapidly grows up to 200 nm, and the size of the nanogap is consequently widened to 200 nm. The widened nanogaps cause not only electrical breakdown, but also leads to physical disconnection of the nanowire.

A schematic to illustrate these phenomena is shown in Fig. 1-c. This sequential process was monitored in real time for the first time with the use of a SEM (Supplementary Video S1). The NSPs and the nanogap were simultaneously formed by the abovementioned electromigration within a time scale shorter than 25 msec. The electrical characteristics that support these phenomena, namely current density (*J*) versus anode voltage (*V*_*A*_), are shown in Fig. 1-d. As the anode voltage increases, the current density also increases, following Ohm’s law. However, after a transition point (3 MA/cm^2^), the slope of the current density becomes steeper. The NSPs and nanogaps are created near the current density of 5 MA/cm^2^, and subsequently permanent physical breakdown, i.e., disconnection of the nanowire, occurs near 6 MA/cm^2^. The inset shows a SEM photograph of the NSPs. The electrical conductance of the nanowire was plotted by differentiating the current with respect to *V*_*A*_, i.e., ∂*I*/∂*V*_*A*_, as shown in Fig. 1-e. As *V*_*A*_ increases, the electrical conductance slowly increases as shown in region I of Fig. 1-e. This phenomenon is well known and understood as the annealing effect of phosphorus^27^,^28^,^29^. When extremely high current flows through the nanowire, Joule heating is concentrated at the middle of the nanowire, as shown in the inset of Fig. 1-e. This heat rapidly accelerates the increase in temperature of the nanowire, and the temperature supplies enough energy to enable the self-activation of dopants. When current density is between 1.5 MA/cm^2^ and 3 MA/cm^2^, a reverse annealing characteristic is triggered, as shown in region II of Fig. 1-e. This reverse annealing characteristic is correlated with dopant solubility and diffusivity. When temperature rises to approximately 600 °C, dopant diffusivity is increased, and diffused dopants form clusters. As a result, the clusters cannot play a role in dopants in silicon lattice, and they are remained at the interstitial sites. Hence, there is a reduced number of carriers and this degrades electrical conductance. This phenomenon has been called “dopant deactivation”, and it can be observed in heavily doped Si^29^,^30^. Beyond the transition point (3 MA/cm^2^), electrical conductance rapidly increases due to further dopant activation. This rapid increase in electrical conductance can be attributed to the enlargement of the grain size in the poly-Si. It is noteworthy that the temperature range that enables dopant activation is overlapped with that needed to trigger the growth in grain size^31^. However, based on transmission electron microscope (TEM) inspection, there was no perceivable change in the grain size below and above the transition point (Figure S2). Beyond the transition point, the nanowire reaches moderately high temperature, which is sufficient to trigger the grain growth. However, there is insufficient time to allow annealing, owing to the integration time needed to measure the current level (see Methods).

In contrast, when the current density was high (5 MA/cm^2^), the short time needed for integration was enough to enable grain growth. Thus the increase in conductance after reaching high current (5 MA/cm^2^) is affected by both dopant activation and grain growth in region III of Fig. 1-e. When the current density is extremely high (6 MA/cm^2^), thermally assisted electromigration is dramatically triggered by the extremely high temperature and the nanowire consequently starts to breakdown.

### Formation of nano silicon particles (NSPs) and mechanism of breakdown

**Figure 2:**
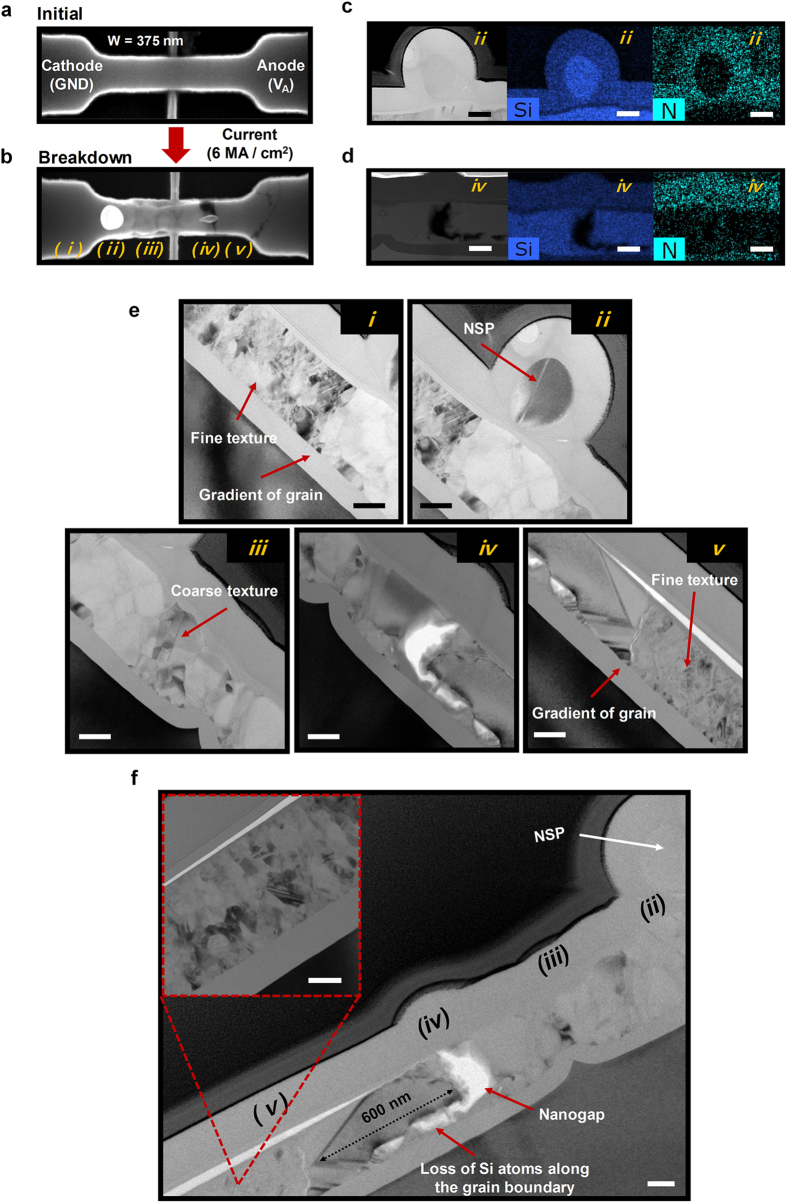
(**a**) SEM image of the initial nanowire without *V*_*A*_. (**b**) Beyond the critical current density (6 MA/cm^2^), breakdown occurs with the creation of the NSP. (ii) and nanogap (iv). (**c**,**d**) TEM and EDS analysis of the NSP and nanogap. (**e,f**) Change of grain size by localized Joule heat. The boundaries of large sized- and small sized-grains determine the location of the NSP (near cathode) and the nanogap (near anode). Scale bar is 100 nm.

Microscopic images of the poly-Si nanowire with the NSPs and nanagaps are shown in Fig. 2. SEM images of the initial nanowire and after breakdown are shown in Fig. 2-a,b. When the current density is larger than 6 MA/cm^2^, NSPs (ii) and nanogaps (iv) are formed simultaneously. The size of the NSP, which initially was smaller than 40 nm, increased up to approximately 200 nm. To analyze these breakdown phenomena precisely, TEM images were taken after an additional Si_3_N_4_ layer of 150 nm thickness was deposited using plasma enhanced chemical vapor deposition (PECVD) at low temperature (150 °C). Moreover, element analysis of the NSP and nanogap was carried out using EDS, and the corresponding results are shown in Fig. 2-c,d. The results confirm that the NSP near the cathode is composed of Si atoms. It has a spherical shape and its crystalline phase is amorphous (Figure S3). However, the inside of the nanowire near the anode is empty due to the migration of Si atoms toward the NSP. In addition, the volume of the NSP and the nanogap are almost the same, as expected. In addition, after breakdown, it can be obviously seen from the cross-sectional TEM images that grain size is varied along the nanowire, as shown in positions (i) to (v) of Fig. 2-e,f. When the grain boundaries are confirmed in advance (Figure S4), it can be seen that the grain size is approximately 600 nm at the middle of the nanowire, and the texture is coarse. In contrast, the grain size of the edge region is about 100 nm, which is similar to the initial grain size of the nanowire before the applied voltage, and the density of texture is fine. These gradients in grain size change are related to heat distribution in the nanowire, which is different at the middle and edges of the nanowire, because the Joule heating is concentrated in the middle of the nanowire (Figures S5 and S6). The NSPs are formed at the boundary where the fine grain texture abruptly changes to the coarse grain texture, while the nanogap is created along the grain boundary where the largest grain texture exists.

### Controllable Electrical and physical breakdown of poly-Si nanowire

**Figure 3:**
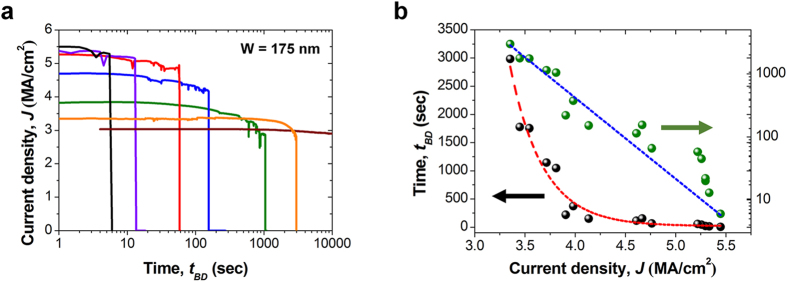
(**a**) Plot of various current densities versus stress time. Black, purple, red, blue, green, orange, and brown represent 5.5 MA/cm^2^, 5.3 MA/cm^2^, 5.1 MA/cm^2^, 4.5 MA/cm^2^, 3.8 MA/cm^2^, 3.2 MA/cm^2^, and 3 MA/cm^2^, respectively. As the current density is increased, *t*_*BD*_ becomes shorter. (**b**) Plot of the extracted *t*_*BD*_ versus the current density from 17 samples. The tendency can be fitted to an exponetial function, which is frequently found in general breakdown phenomena.

An interesting fact was observed the location of the NSPs and the nanogap are dependent on the flow direction of the current. The NSPs are always formed near the cathode and the nanagap is created near the anode, as mentioned above. If the current direction is intentionally reversed, the locations of the NSPs and nanogap are also transposed. This fact was carefully confirmed through more than 100 extra measurements. This dependency of the NSPs and the nanogap on current direction is related to the grain size change of the nanowire^32^. There are many grain boundaries in the fine textured regions. However, there are few grain boundaries in the coarse texture regions. Therefore, the movement of Si atoms is much easier in the fine texture than the coarse texture. When the electron wind produced by extremely high current flows from the cathode to the anode, Si atoms also move toward the anode. At the interface between different grain textures near the cathode, the number of grain boundaries abruptly decreases, and Si atoms are piled up near the cathode. These accumulated Si atoms aggregate in the form of the NSPs. By the same process, the migration of Si atoms from near the anode leaves the nanogap. Through this mechanism, the poly-Si nanowire experiences a catastrophic and morphic change under high current density. As a consequence, the formation of the NSPs and nanogaps leads to the nanowire breaking. Electrically, the NSPs can cause a short circuit problem, or the nanogap can cause an open circuit breakdown. To prevent the aforementioned high current-induced problems in advance, the relationship between current density versus time-to-breakdown (*t*_*BD*_) should be clarified. *t*_*BD*_ was measured for various constant current densities, and the results are shown in Fig. 3-a,b. As the current density (*J*) increases, *t*_*BD*_ decreases exponentially, i.e., *t*_*BD*_ ∝ exp(–*BJ*), which is also frequently found in general breakdown phenomena elsewhere. Notably, at a current density level of 3 MA/cm^2^, which is close to the transition point, there was no indication of any electromigration even after 12 hours. This absence of electromigration is ascribed to the relatively low temperature and weak electron wind from the low current density.

### Size and shape of the NSPs and nanogap

**Figure 4:**
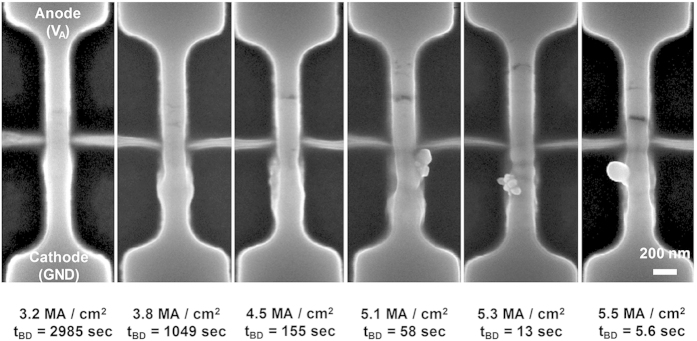
SEM images of nanogap and NSPs with various current densities. The size and shape of the NSPs depend on the current density. When electromigration occurs with high current and short time, the shape of the NSPs is close to a sphere.

**Figure 5:**
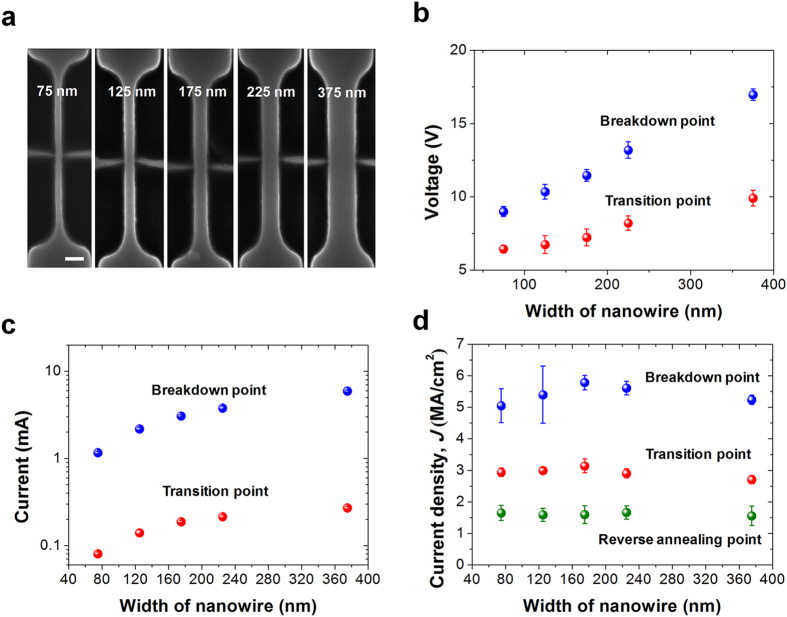
(**a**) SEM images of various nanowire widths between 75 nm and 375 nm. (**b,c**) Relationship between the voltage and current versus the nanowire width. (**d**) Relationship between the three normalized critical current (Reverse annealing point, transition point, breakdown point) versus the nanowire width. The critical current density is nearly independent of the nanowire width. Electrical and thermal characteristics are reproducible and controllable regardless of the nanowire width. 18 samples were measured for each dimension and standard deviation was used for the error statistics. Scale bar is 200 nm.

The size of the NSPs depends on the current density, and this relationship is shown in Fig. 4. Under high current conditions, the shape of the NSP is close to a sphere, and the overall height of the NSP tends increase as a function of the current density. Above 5.5 MA/cm^2^ the size of the NSPs is close to 200 nm, and this can cause an electrical short between the adjacent interconnection lines. In turn, under the low current condition, the nanowire begins to swell, but without the formation of the NSPs. Since high current density generates a much higher temperature than low current density, the grain size and texture are more abruptly changed under the high current condition, thus causing a catastrophic change, such as NSPs formation, to occur. Consequently, the relationship between the current density, *t*_*BD*_, temperature, and line-to-line spacing should be carefully analyzed, and those parameters must be properly engineered. Many nanowires were fabricated with various widths to confirm whether these extremely high current density-induced properties are controllable and reproducible. SEM images of the fabricated nanowires are shown in Fig. 5-a. As denoted in Fig. 1-e, there are three critical current densities: at the reverse annealing point (1.5 MA/cm^2^) which produces a region of increasing resistance; the transition point (3 MA/cm^2^) which results in increased electrical conductance; and at the breakdown point (6 MA/cm^2^) which causes the nanowire breakage. The critical voltage and current needed to change the state of the poly-Si nanowire increases according to increases in the nanowire width, as shown in Fig. 5-b,c. But, when the current is normalized by a cross-sectional conduction area, current density is almost constant regardless of the nanowire width as shown in Fig. 5-d. Hence, the critical point needed to trigger the reverse annealing, phase transition, and breakdown is independent of the nanowire width, as expected. From this it can be inferred that physical and electrical breakdown are controllable, and thus reproducible, regardless of the nanowire width.

Simulations of the temperatures of the reverse annealing point and transition point determined that they were approximately 160 °C, 600 °C, respectively, and those temperatures were confirmed through two kinds of simulation (Figures S5 and S6). When modeling is conducted using those results, *t*_*BD*_, governed by the thermally assisted electromigration of poly-Si, can be predicted, and thus device failure can be avoided.

## Discussion

The controllability of the electrical and physical breakdown of poly-Si nanowires was demonstrated, and the underlying physics was determined to be thermally assisted electromigration. These results explain the formation of the NSPs and nanogaps, which are simultaneously created via the thermally assisted electromigration. Interestingly, the NSPs are always formed near the cathode, while the nanogaps were created near the anode. For the first time, catastrophic and morphic changes of a poly-Si nanowire were observed with real time monitoring, by using a SEM. The morphic changes of the poly-Si nanowire could be categorized into three parts: a reverse annealing region, a transition point with increasing electrical conductance, and a breakdown point resulting in the breakage of the nanowire. By controlling the critical current density to distinguish the three regions, the electrical and physical breakdown were artificially controlled, and the reproducibility of control of the abovementioned breakdown was also confirmed. It was also experimentally proved that the critical current density governing the phase states of the poly-Si nanowire did not vary with the nanowire width. This work can pave the way to understanding the breakdown phenomena of poly-Si nanowires and provide a powerful tool for intentionally engineering the phase of the poly-Si.

## Methods

### Fabrication of the poly-Si nanowire

A top down method based on photo-lithography and etching was used to precisely fabricate poly-Si nanowire patterns^9^,^12^,^33^. To begin, a 70 nm thickness of tetraethyl orthosilicate (TEOS) was deposited on a p-type silicon substrate using low pressure chemical vapor deposition (LPCVD), and an *in-situ* phosphorus doped poly-Si film of 300 nm thickness and oxide hard mask (passivation) layer of 30 nm were deposited by LPCVD. After photo-lithography, partial ashing with the use of O_2_ plasma was performed to reduce the nanowire width, and then the poly-Si film was etched. Afterwards, the remaining photo-resist (PR) was completely removed. For dopant activation, rapid thermal annealing (RTA) at 1000 °C for 3 sec was used. The width of the fabricated nanowires were in the range of 75 nm to 375 nm, and length was fixed at 2 μm.

### Electrical measurement system

A semiconductor parameter analyzer (HP 4155B) was used to measure DC characteristics under ambient air at room temperature. Its integration time and voltage step were set to 16.7 msec and 500 mV, respectively. Vacuum measurements were also performed under 1 mTorr, to confirm the influence of ambient oxygen. However, the oxygen induced no effects due to the oxide passivation layers of 30 nm thickness.

### Structural characterization

The SEM images were taken using a FE-SEM manufactured by FEI, and its accelerating voltage was 10 kV. The JEM-ARM200F system manufactured by JEOL was used for the TEM and EDS analysis with 200 kV accelerating. For real time SEM inspection, the FEI Quanta 3D FEG was utilized under 5 kV. Frames of the recorded video images were 42 frames per sec.

## Additional Information

**How to cite this article**: Park, J.-Y. *et al.* Controllable electrical and physical breakdown of poly-crystalline silicon nanowires by thermally assisted electromigration. *Sci. Rep.* **6**, 19314; doi: 10.1038/srep19314 (2016).

## Supplementary Material

Supplementary Information

Supplementary Video S1
