# Supplementary material for: Controllable electrical and physical breakdown of poly-crystalline silicon nanowires by thermally assisted electromigration
Source: Sci Rep. 2016 Jan 19;6:19314. doi: 10.1038/srep19314 (PMC4726027; doi:10.1038/srep19314)
Supplement: Supplementary Information [file srep19314-s1.pdf]

Supplementary Information

Controllable electrical and physical breakdown of poly-crystalline silicon nanowires by thermally assisted electromigration

Jun-Young Park,[1] *Dong-Il Moon,*[1] *Myeong-Lok Seol,*[1] *Chang-Hoon Jeon,*[1,2] *Gwang-Jae Jeon,*[1] *Jin-Woo Han,*[3] *Choong-Ki Kim,*[1] *Sang-Jae Park,*[1] *Hee Chul Lee,*[1] *and Yang-Kyu Choi*[1]*

[1]School of Electrical Engineering, Korea Advanced Institute of Science and Technology (KAIST), Daejeon 34141, Republic of Korea

[2]Semiconductor R&D Center, Samsung Electronics, San #16 Banwol-Dong, Hwasung-City, Gyeonggi-Do 445-701, Republic of Korea

[3]Center for Nanotechnology, NASA Ames Research Center, Moffett Field, CA 94035, USA

* Address correspondence to ykchoi@ee.kaist.ac.kr

Initiating thermally assisted electromigration and creating nano silicon particles (NSPs)

As the anode voltage (V_A) increases, the current density is increased. When the current density reaches 5 MA/cm^2, a nanogap and NSPs start to form with the rise in current. Electrical measurement results obtained at this stage are shown in Figure S1a. The size of the NSP which is formed at this current density is tiny compared with the larger sized NSP formed with catastrophic failure at 6 MA/cm^2. The diameter of the single NSP is approximately 40 nm, and is created on the surface of the nanowire near the cathode. This tiny single NSP is considered to be the seed of the larger NSP, because it grows up to 200 nm after failure. At the current density of 5 MA/cm^2, a nanogap starts to form near the anode, simultaneously with the growth of the NSP. Figure S1b shows the SEM image of this stage of nanowire. The top electrode is assigned as the anode and the bottom electrode is assigned as the cathode. Figure 1Sc shows a magnified SEM image of the nanowire used to investigate the initial stage of NSP formation. The shape of the NSP is spherical, and the diameter is approximately 40 nm. At the beginning of this research, we thought that this tiny sphere structure came from vaporized silicon atoms due to its spherical shape. However, this NSP is not related to melted and evaporated silicon.

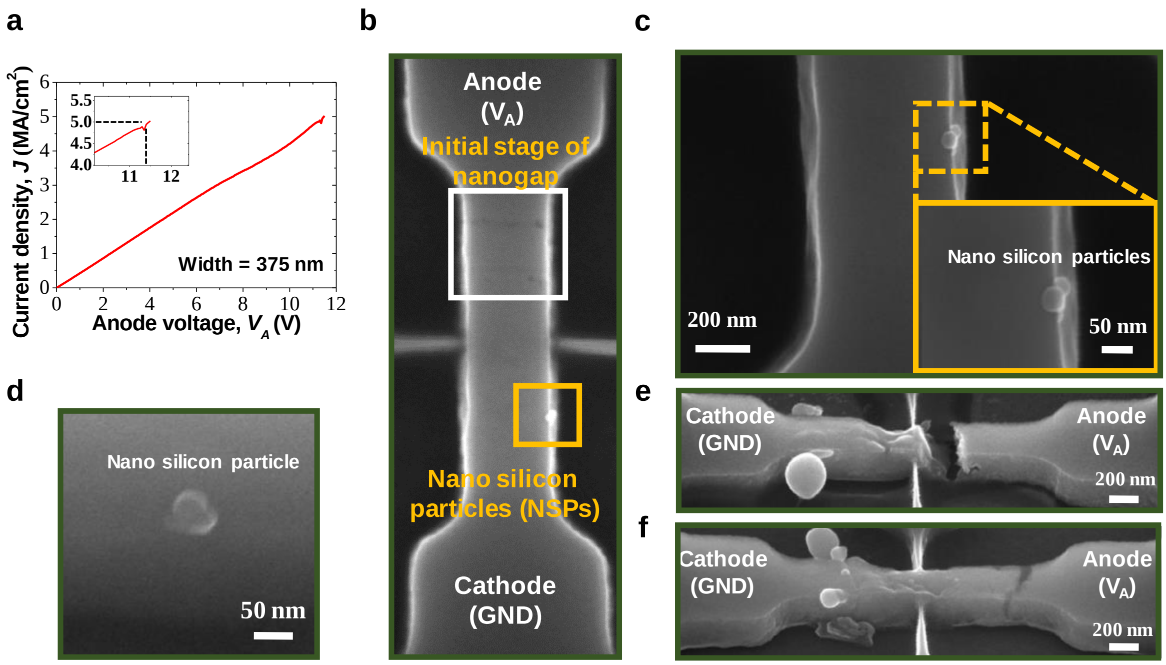

Figure S1. Initial formation of nanogap and NSP, and its mechanism. a) Electrically measured data of applied voltage up to 5 MA/cm^2, when the initial stages of the nanogap and NSP start to form. b) SEM image of nanowire with low magnification. When the current density reaches 5 MA/cm^2, the initial stages of the nanogap and NSP start to form near the anode, and cathode, respectively. c) SEM image of the initial stage of the NSP under high magnification. d) Even after the oxide passivation layers are etched away, the NSP is not removed. This fact means that the NSP protrudes from the surface of the poly-Si. e) SEM image of wet etched nanogap and NSP, which were formed with catastrophic failure at 6 MA/cm^2. f) SEM image of catastrophic biased nanowire, which does not have oxide passivation layers.

In order to confirm the mechanism of the NSP formation, 30 nm of oxide passivation layers were removed using buffered oxide etchant (BOE). If the mechanism of catastrophic failure was related to the melting, evaporation and re-solidification of silicon atoms, the NSP should be etched away. Even after sufficient etching was performed for 2 min, the tiny NSP

was not removed at all, and this fact confirms that in its initial stage the NSP starts to form on the surface of the poly-Si, and it grows and rises further after penetrating the 30 nm of oxide passivation layers. Figure S1d shows the SEM image of the initial stage NSP after removal of the oxide passivation layers. And Figure S1e shows the NSP which was formed with the catastrophic failure at 6 MA/cm^2, and after the oxide passivation layers were etched using BOE. The location of the nanogap and NSP were also confirmed through an extra experiment, and the formation of those structures was not affected by the existence of oxide passivation layers; the result is shown in Figure S1f. The initial nanowire without applied bias V_A was etched to remove the oxide passivation layers using BOE. Then, catastrophic voltage was applied to the nanowire to confirm the effect of the oxide passivation layers, with the result that the existence of the oxide passivation layers were not related to nanogap and NSP formation. The nanogap and NSP were still formed relative to the anode and cathode, respectively.

Crystalline phase of nanowire and NSP, and confirmation of grain boundary

The increase in electrical conductance in region III has two possible causes: dopant activation and grain growth. The dopant activation phenomena has been reported to occur at temperatures much higher than 700 °C, and grain growth of poly-Si can happen at a temperature much higher than 800 °C[1,2]. So, the temperature ranges of dopant activation and grain growth are overlapped. We performed an experiment to verify which was the dominant factor in the increase of electrical conductance in region III. For that analysis, TEM was used and the results are shown in Figure S2.

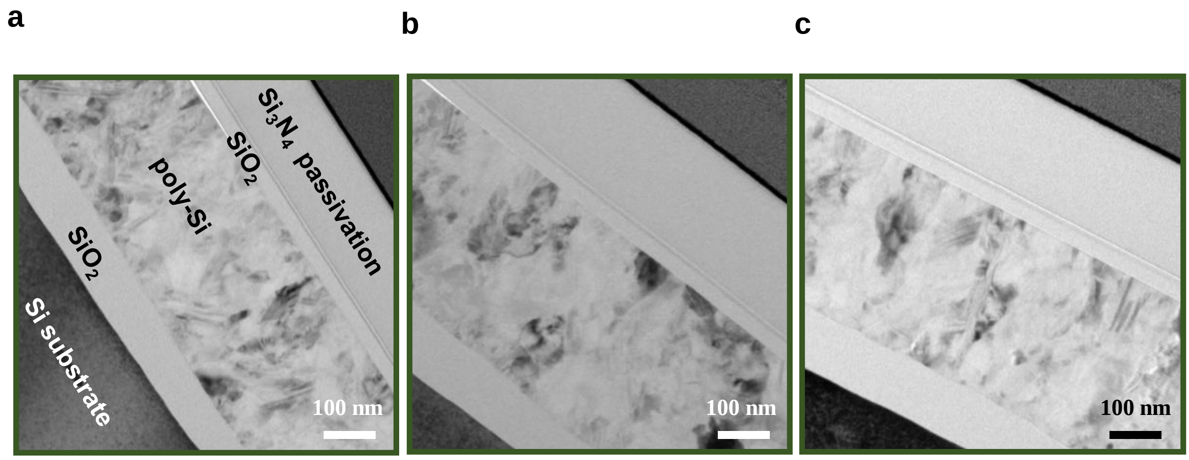

Figure S2. Cross-sectional TEM images of the nanowire before and after the transition point. (a) Without applied bias V_A. (b) At transition point (3 MA/cm^2). (c) After transition point (3.8 MA/cm^2).

Before and after the transition point (3 MA/cm^2), there was no perceivable change in the grain texture. This fact means that grain growth was not a major factor in the increasing electrical conductance, and dopant activation was therefore the dominant factor. The grain growth phenomena depends on the annealing time, meaning that both high temperature and enough time are needed[1]. On the other hand, dopant activation happens very fast compared

with grain growth. We used 16.7 msec of integration time for the measurement, and this time was not enough to cause grain growth at 3 MA/cm^2. However, if enough time is given, grain growth can happen. In this respect, it is also clear that when an extremely high current density is applied, even a very short annealing time can change the grain texture. The NSP is composed of silicon atoms and grew from the tiny, so-called initial stage of the NSP, which started to form at 5 MA/cm^2. The phase of the NSP can be identified using TEM, and the image is shown in Figure S3. Grain boundaries should also be identified to confirm the change of grain size, as this is correlated with the mechanism of catastrophic failure. Figure S4a, b show cross-sectional TEM images of the poly-Si nanowire, and grain boundaries can be confirmed.

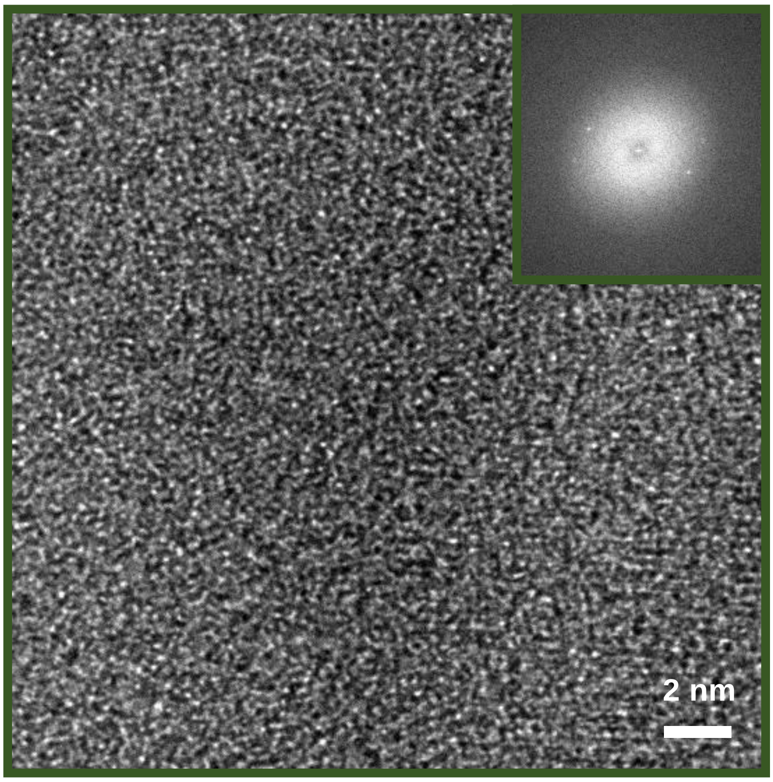

Figure S3. A TEM image of a NSP under high magnification. A cross-sectional image of a NSP which was grown at the failure current density (6 MA/cm^2) was cut for phase analysis. The phase of the NSP was revealed to be amorphous.

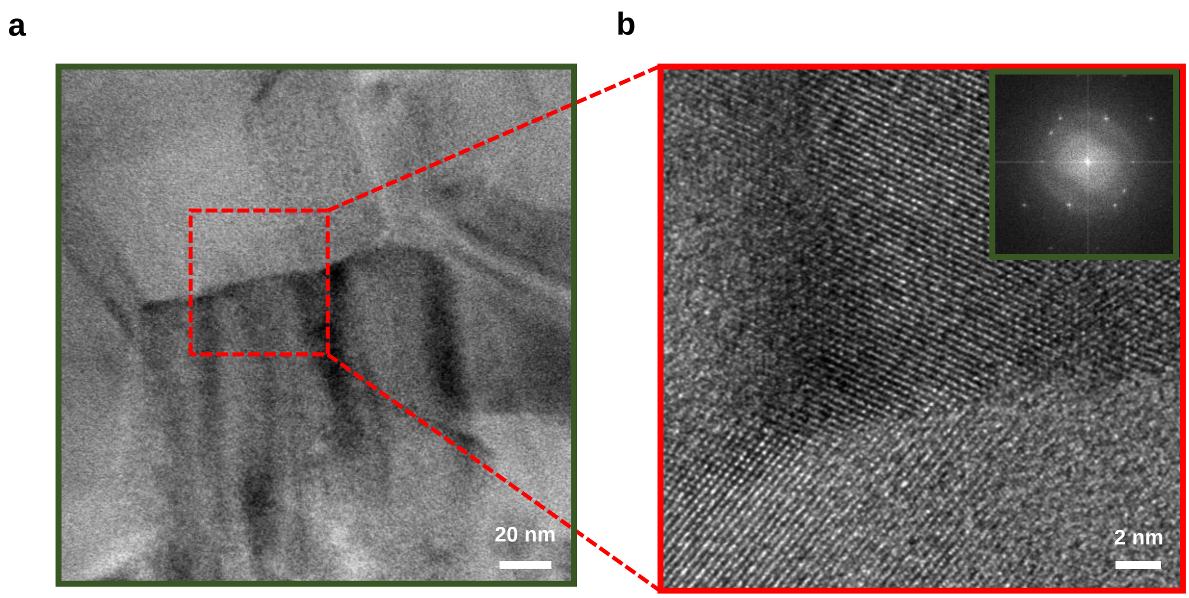

Figure S4. Confirmation of grain boundaries for grain size gradient. a) Grain boundaries with low magnification TEM analysis. b) Verification of grain boundaries using high magnification TEM analysis.

Numerical simulation for understanding heat distribution

Heat distribution is the most important factor in this research. Numerical simulations were used to understand the heat distribution of a poly-Si nanowire. Two kinds of well-known simulation tools, COMSOL and ANSYS, were used to validate the simulation results. All conditions including design and material parameters were the same for each simulation. The thermal conductivities of poly-Si and SiO_2 were used with reference to previous studies[3,4]. The most important parameter was resistivity, and it was obtained by extracting the real measurement data in Figure 1d for each bias (V_A).

Figure S5a shows a top-view of the device structure including two electrodes, and a cross-sectional view of the nanowire is shown in Figure S5b with design parameters. Most of the heat energy is concentrated at the middle of the nanowire, and grain size changes with heat distribution in the nanowire. This heat distribution profile is shown in Figure S5c, d. The temperature of the nanowire was also quantitatively analyzed using those simulations. As voltage (V_A) increases, the temperature of the nanowire is also increased, and this is plotted in Figure S5e. The heat distribution profile and temperature analysis of the nanowire that were conducted by the two different kinds of tools were the same. Then, this simulated temperature was combined with the electrical measurement data shown in Figure 1d. Consequently, the temperature versus current density was found and is shown in Figure S5f.

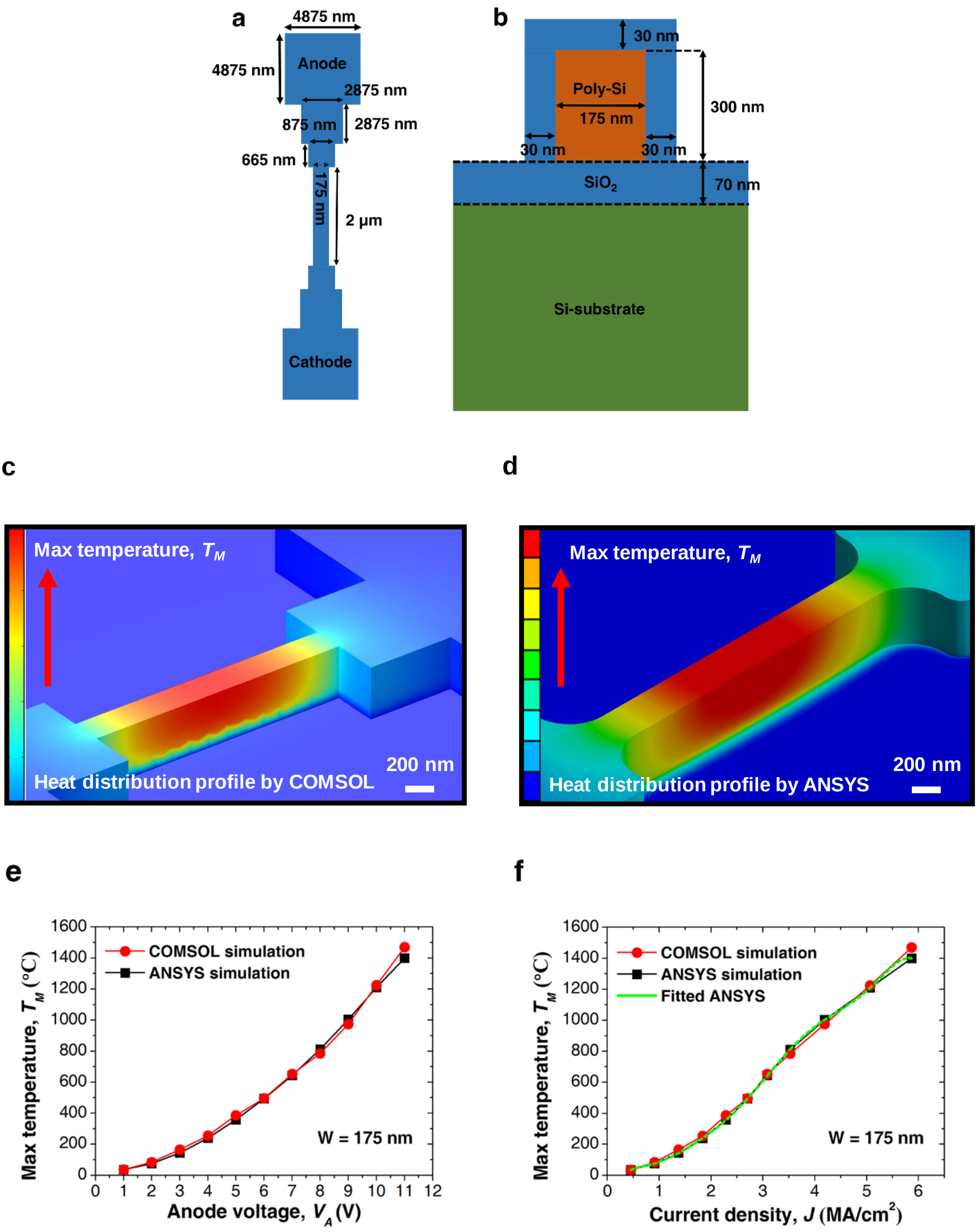

Figure S5. Simulation results for the heat distribution and quantitative analysis of temperature. a) Top-view of the nanowire including electrodes which were used for the simulation. b) Cross-sectional view of the silicon nanowire with design parameters. c) Heat

distribution profiles of the nanowire using COMSOL and d) ANSYS. e) Extracted temperature with increasing anode voltage. Those results correspond with each other. f) Temperature of nanowire versus current density. The two simulation tools show a similar temperature range.

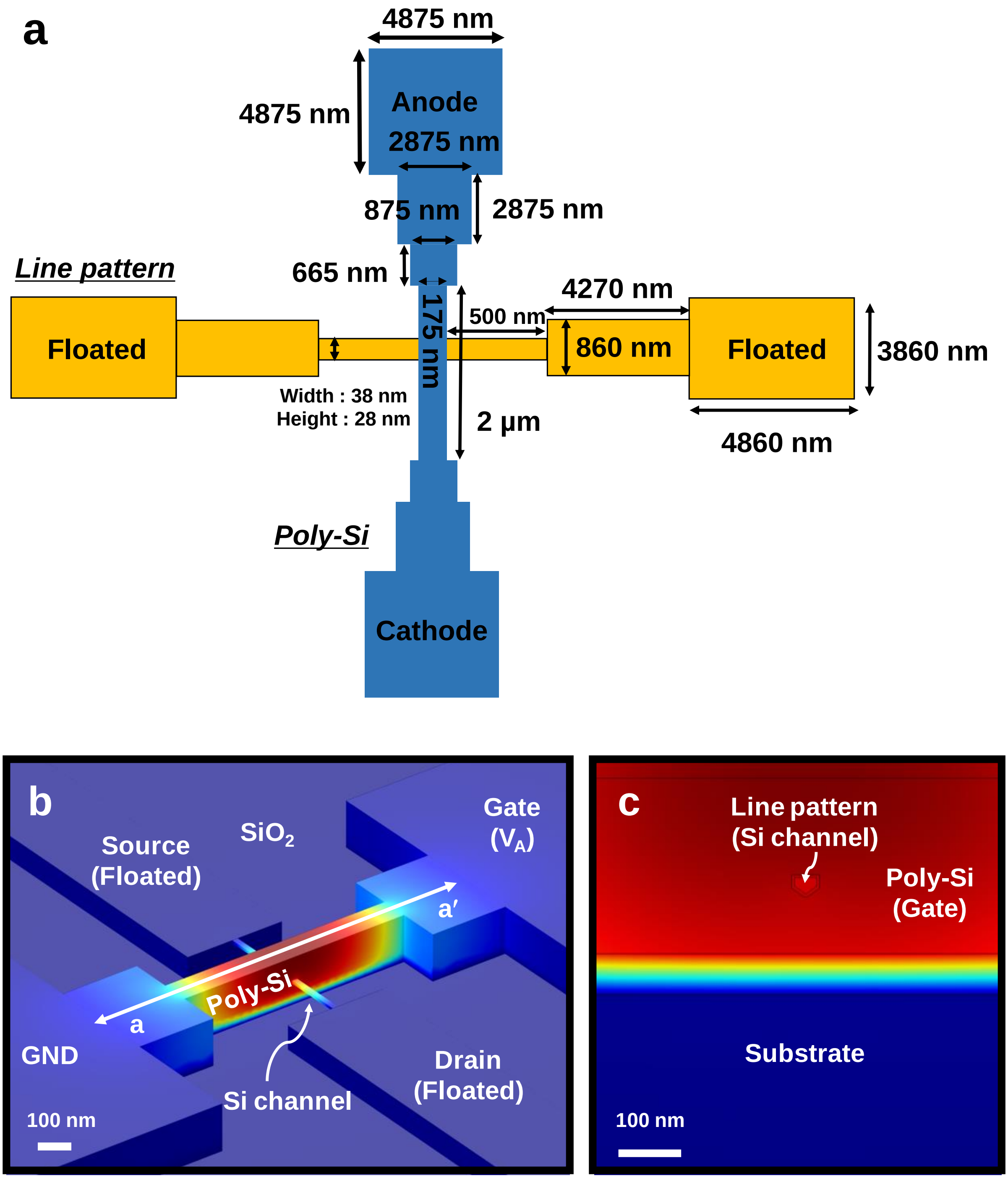

d

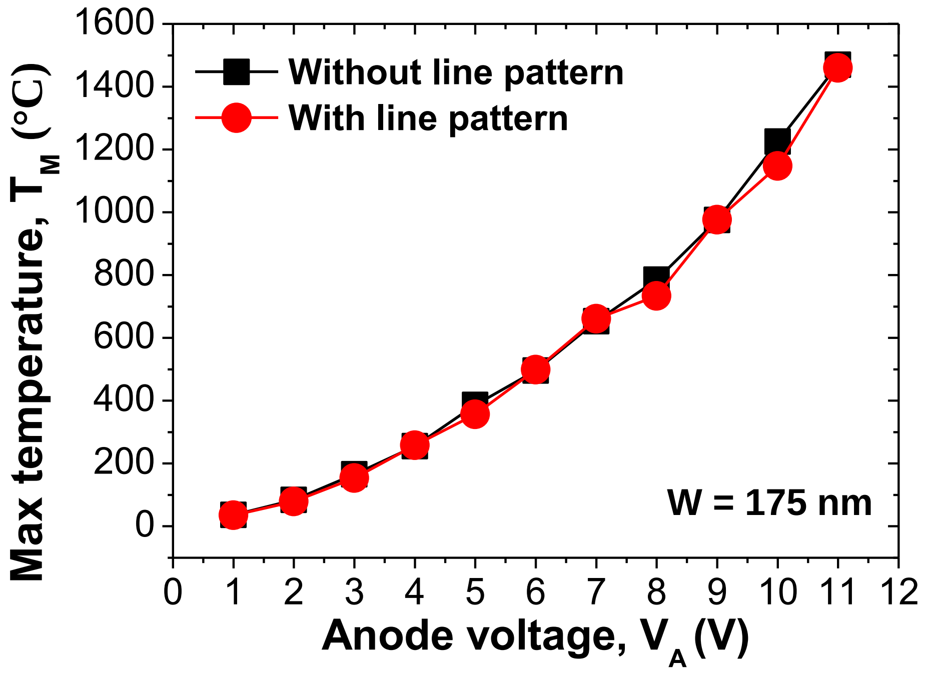

Figures S6. Influence of the heat distribution profile with an underlying line pattern (Si-channel), which exists across the poly-Si nanowire. a) Top-view of the poly-Si nanowire including the underlying line pattern, which has two floated electrodes: source and drain in a MOSFET. b) Simulated heat distribution profile in the poly-Si nanowire. c) Cross sectional image of the heat profile along a-a' direction in Figure S6-b. d) Calculated temperature with existence of the underlying line pattern. When the underlying line pattern is included, the created temperature is decreased by the amount of approximately 15 °C per each voltage step, which is ignorable.

1. Wada, Y. Grain Growth Mechanism of Heavily Phosphorus-Implanted Polycrystalline Silicon. *J. Electrochem. Soc.* **125,** 1499 (1978).

2. Bicknell, R. W. The annealing characteristics of phosphorous implanted silicon. I. *Philos. Mag.* **26,** 273–286 (1972).

3. Xie, J., Lee, C., Wang, M.-F., Liu, Y. & Feng, H. Characterization of heavily doped polysilicon films for CMOS-MEMS thermoelectric power generators. *J. Micromechanics Microengineering* **19,** 125029 (2009).

4. Shackelford, J. F. and Alexander, W. Materials Science and Engineering Handbook 3rd edn, Ch. 5, 396-552 (CRC, 2001).

Video Legend

A video file is attached as supplementary information file.

Video S1. Real time observation of thermally assisted electromigration.
